# Use of probiotics in preventing and treating excess weight and obesity. A systematic review

**DOI:** 10.1002/osp4.759

**Published:** 2024-06-19

**Authors:** Belén Torres, María C. Sánchez, Leire Virto, Arancha Llama‐Palacios, María J. Ciudad, Luis Collado

**Affiliations:** ^1^ Faculty of Medicine Department of Medicine Complutense University Madrid Spain; ^2^ Faculty of Medicine GINTRAMIS Research Group (Translational Research Group on Microbiota and Health) Complutense University Madrid Spain; ^3^ Faculty of Optics Department of Anatomy and Embryology Complutense University Madrid Spain

**Keywords:** *Bifidobacterium*, *Lactobacillus*, obesity, overweight, probiotics

## Abstract

**Background:**

The prevalence of excess weight and obesity is increasing in an extremely concerning manner worldwide, with highly diverse therapies for current treatment. This review evaluated the scientific evidence of the past 10 years on the use of probiotics in treating excess weight and obesity in the absence of dieting.

**Materials:**

A systematic review was conducted by searching for clinical trials on humans published in English in the PubMed, Scopus and Cochrane Central databases, using the combination of keywords “Overweight”, “Probiotics” and “Obesity”, and published between 2012 and 2022.

**Results:**

Six published studies met the inclusion criteria. The review showed that, although there is a lack of consensus in the literature, the use of probiotics in the absence of dieting produced a significant reduction in body weight and body mass index in 66.6% of the reviewed studies, a significant reduction in waist circumference in 80.0% of the reviewed studies, and an improvement in total body fat mass and waist circumference.

**Conclusions:**

This review showed evidence of a trend in preventing body weight gain and reducing weight through the use of probiotics in individuals with excess weight or obesity. A combination of various strains of the genera *Bifidobacterium* and *Lactobacillus* was the most effective.

## 1 INTRODUCTION

Located mainly in the colon, the human gut microbiota is one of the most complex ecosystems colonizing the human body, comprising hundreds of species and composed mostly of anaerobic bacteria.^1^, ^2^ There is significant microbial diversity among humans at the species and strain levels, with each individual harboring their own distinctive bacterial composition, determined among other factors by the host's genotype, the initial colonization at birth via vertical transmission, and dietary habits.^1^, ^2^ In this ecosystem, the two predominant bacterial divisions are *Bacteroidetes* and *Firmicutes*, accounting for more than 90% of microbes, followed by *Actinobacteria*, *Proteobacteria*, *Verrucomicrobia*, and *Fusobacteria*.^3^, ^4^

The relatively stable gut microbial community regulates intestinal homeostasis, providing metabolic, protective, and structural benefits to the intestinal epithelial cells and demonstrating extra‐intestinal effects by establishing a communication axis among the organs via neural, endocrine, immune, humoral, and metabolic pathways.^5^ Gut microbiota play a significant role in digestion, vitamin synthesis, metabolic processes (e.g., the regulation of cholesterol absorption, blood pressure, and glucose metabolism), and protection against pathogens, while also being involved in central nervous system modulation, host immune modulation and maturation, host development and physiology (organ development), differentiation and proliferation of intestinal epithelium and intestinal angiogenesis.^6^, ^7^, ^8^

This microbial consortium has its own homeostatic capacity, which gives stability to this ecosystem and allows a symbiotic relationship with the host, which however, on occasions and due to various factors, can be overcome, causing dysbiosis of the microbiota with the consequent impact on intestinal homeostasis. Gut microbiota disorders have been implicated in various forms of inflammatory bowel disease (including Crohn's disease and ulcerative colitis^9^, ^10^, ^11^, ^12^, ^13^, ^14^), atopic asthma, behavioral disorders, obesity, type 2 diabetes mellitus, cardiovascular diseases, and autoimmune disorders.^15^ In this regard and to maintenance of normobiosis, the beneficial effects of probiotic bacteria are becoming increasingly evident, and numerous scientific reports have confirmed probiotics' positive effects on gut microbiota and thus on the host's health in a variety of conditions, including obesity, insulin resistance syndrome, type 2 diabetes mellitus, and non‐alcohol fatty liver disease.^15^, ^16^, ^17^, ^18^

Human intestinal probiotics are microorganisms that provide a health benefit when applied in appropriate doses, demonstrating significant effectiveness in maintaining intestinal health, and thereby general health, counteracting diseases triggered by intestinal disorders.^19^, ^20^ Several studies show that probiotics might be useful in treating various gastrointestinal disorders, types of diarrhea, irritable bowel syndrome, enteritis, and bacterial infections. However, there is currently a broad debate on the influence of probiotics on the body's metabolism especially regarding obesity.

Obesity is considered a chronic disease due to its health risks and the biological mechanisms that impede weight loss.^21^ Short‐term interventions, either behavioral or medical, are usually insufficient to achieve lasting weight loss. Although promoting a healthy diet and more physical activity is important for preventing obesity, these measures are insufficient for reducing the body mass index (BMI) of individuals who already have a high weight.^22^ New treatment strategies have therefore been developed in recent years. In addition to the classical treatment options, such as glucagon‐like peptide‐1 receptor agonists,^23^ research has focused on the microbiome as a possible target for fighting obesity.^24^, ^25^, ^26^

Recent studies, both in humans and in animal models, have shown that the intestinal microbiota affects the onset of obesity and governs the body's metabolic functions.^17^ Additionally, experimental models have shown that certain bacterial strains can inhibit or attenuate the immune responses associated with chronic inflammation.^16^ Studies have discovered that various genera of microorganisms, such as *Lactobacillus*, *Bifidobacterium*, *Saccharomyces*, *Streptococcus* and *Enterococcus*, can play a role in preventing or managing obesity through their supplementation in the diet.^27^, ^28^, ^29^, ^30^, ^31^

Probiotics have been observed to influence the control of obesity through various mechanisms of action such as regulating the functions of endogenous microbiota. Studies have also observed that probiotics compete with pathogens, improve intestinal barrier function and strengthen the natural immune responses. There is currently a broad debate on the influence of probiotics on the body's metabolism especially regarding obesity,^18^ and there are conflicting data and a lack of knowledge about the long‐term effects.^32^ Additionally, probiotic activity might depend on the strain, dosage, and components employed to produce a given probiotic product. The objective of this review was therefore to summarize the main effects of probiotics in preventing and treating excess weight and obesity, highlighting the most recent developments in their clinical application and focusing on the employed strain.

## 2 MATERIALS

### 2.1 Study design

The present review followed the criteria of the PRISMA declaration (Preferred Reporting Items for Systematic Reviews and Meta‐Analyses).^33^ The study was designed to answer the following specific question: Is treatment with probiotics effective in patients with excess weight or obesity? The study applied the PICO nomenclature [P (population): adult individuals with excess weight and obesity; I (intervention): treatment with probiotics in the absence of diet; C (Comparison): comparison of 2 groups, one treated and one given placebo; O (Outcomes): weight loss, reduction in BMI, waist‐hip index (WHI) and waist circumference].

### 2.2 Search strategy for scientific evidence

The scientific literature search was conducted in January 2022 in the Medline/PubMed, Scopus and Cochrane CENTRAL databases, encompassing the last 10 years (2012–2022). The keywords employed were “Overweight”, “Probiotics” and “Obesity” combined with the Boolean operator “AND”. The criteria employed for diagnosing obesity and excess weight in the included studies could be any of the following: weight, BMI, WHI and waist circumference.

### 2.3 Selection of studies and data collection. Eligibility criteria

The eligibility evaluation was conducted by analyzing the title, abstract and full text. Three reviewers independently selected titles and abstracts for possible inclusion in the review according to the inclusion criteria, which are detailed below. If discrepancies appeared, they were resolved by another three independent reviewers. The inclusion criteria were the following: clinical trials and randomized controlled trials performed with humans, patients with excess weight or obesity, age of 18–65 years, published in English and in the last 10 years (2012–2022), and those related to the event of interest. The exclusion criteria were as follows: participants with associated medical conditions (including hypertension, diabetes, metabolic syndrome, Prader‐Willi syndrome, cancer), pregnant participants, participants younger than 18 years or older than 65 years, trials published in languages other than English, participants who underwent additional long‐term or sporadic medical treatment, and participants who were dieting and/or supplementing with symbiotics, natural probiotics (yogurt, kefir), or additional substances.

### 2.4 Information extraction

The information was extracted by three reviewers under the supervision of three other independent reviewers.

### 2.5 Risk of bias assessment

Three reviewers assessed the quality of the included articles using the checklist of the Joanna Briggs Institute (*JBI Manual for Evidence Synthesis*. JBI, 2020. Available from https://synthesismanual.jbi.global). This tool evaluates randomized clinical trials with respect to 13 domains that assess the methodological quality of each trial.

## 3 RESULTS

### 3.1 Selection process for the articles of interest

**FIGURE 1:**
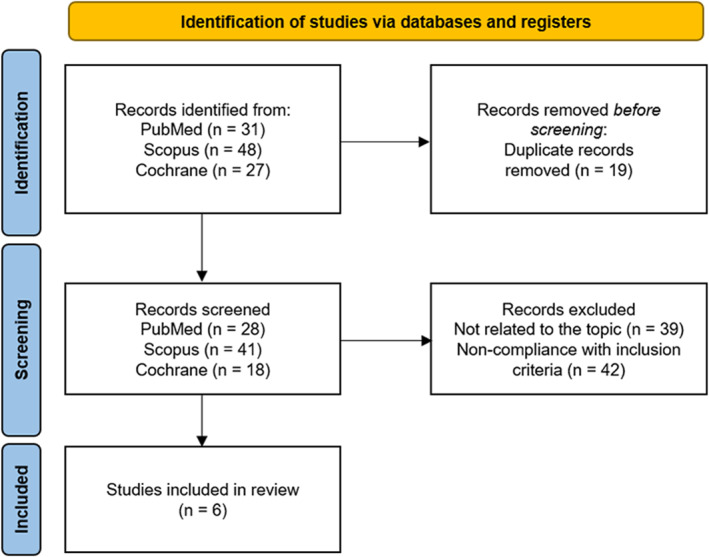
Flowchart of selected articles.

The flow diagram is shown in Figure 1. The search was conducted in January 2022 in three databases (Medline/PubMed, Scopus and Cochrane CENTRAL) using a series of filters based on the established inclusion and exclusion criteria and available in the database, as indicated below. In the PubMed database, the following filters were employed: Text availability (Free full text); Article type (clinical trial and randomized controlled trial); Publication date (10 years), Species (humans) and Age (19–64 years), obtaining a total of 31 results. In the Scopus database, the following filters were employed: Open Access (all open access); Keywords (Obesity AND Overweight AND Probiotics), obtaining a total of 48 results. In the Cochrane Central database, the following filters were employed: Custom Range (2012–2022) and Trials, obtaining a total of 27 results. In total, the search resulted in 106 articles, 19 of which were duplicates, leaving 87. After the titles and abstracts were reviewed, six articles that met the established inclusion criteria were ultimately selected, while 77 were excluded for various reasons, 39 of them for not rigorously answering the proposed question or for not assessing the parameters of interest, and the rest for not meeting the eligibility criteria described above: medical conditions in the study population (*n* = 10), supplementation with symbiotics (*n* = 6), natural probiotics (*n* = 3), additional substances (*n* = 3), pregnant participants (*n* = 8), not clinical trials (*n* = 7), additional medical treatment (*n* = 1), performed in animals (n = 1) or age outside the determined range (*n* = 3).

### 3.2 Results of the risk of bias assessment

**FIGURE 2:**
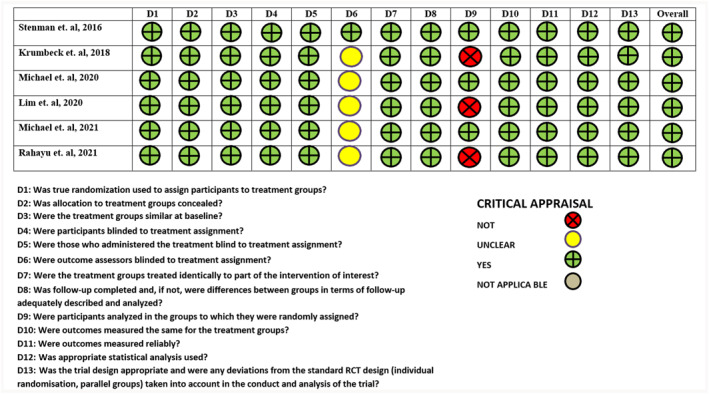
Assessment of the risk of bias of the articles included in the systematic review.

The JBI checklist was employed to determine the risk of bias, obtaining a traffic plot graph (Figure 2) where the risk of bias for each article selected for this purpose could be clearly observed. None of the articles had three or more high‐risk judgments, and all were included in the review.

### 3.3 Characteristics of the studies

The main characteristics of the studies included in the systematic review are shown in Table 1.

**TABLE 1:** Main characteristics of the study population, studied Probiotic, and dosage and trial duration.

No	Author and year	Study design	Study population			Baseline measurements	Probiotic studied	Dosage	Consump‐tion method	Treatment duration
			Treatment		Placebo					
1	Stenman et. al, 2016^37^	Clinical trial randomized by blocks, double‐blind	*n* = 25		*n* = 36	BMI 28–34.8 kg/m^2^	*Bifidobacterium animalis* ssp. *lactis* 420 (B420)	1 × 10^10^ cfu/day	1 small bag daily mixed with 250 mL of fruit milkshake	24 weeks
			Age (mean) in years 49.1		Age (mean) in years 48.3	WHI ≥ 0.88 (men)				
			72% women		72.2% women	WHI ≥ 0.83 (women)				
2	Krumbeck et. al, 2018^36^	Double‐blind, parallel‐arm, randomized clinical trial	*n* = 14 (IVS‐1)	*n* = 14 (BB‐ 12)	*n* = 17	BMI 30–40 kg/m^2^	*Bifidobacterium adolescentis* (IVS‐1)	1 × 10^9^ cfu/day	1 small bag daily mixed with a bottle of water (allowing 2 h to pass before eating food)	3 weeks
			Age (mean) in years 44.7	Age (mean) in years 43.9	Age (mean) in years 43.9		*B*. *animalis* ssp. *lactis* (BB‐12)			
			64.3% women	75% women	76.5% women					
3	Michael et. al, 2020^39^	Single‐center, double‐blind, parallel‐arm, randomized clinical trial	*n* = 110		*n* = 110	BMI 25–34.9 kg/m^2^	*Lactobacillus acidophilus* CUL60 (NCIMB 30157)	5 × 10^10^ cfu/day	Ingestion of 1 capsule daily along with food, with or without a cold beverage	24 weeks
							*L*. *acidophilus* CUL21 (NCIMB 30156)			
			Age (mean) in years 45.30		Age (mean) in years 46.52	Waist circumference ˃89 cm (women)	*Lactobacillus plantarum* CUL66 (NCIMB 30280)			
							*Bifidobacterium bifidum* CUL20 (NCIMB 30153)			
			60% women		60.9% women	Waist circumference ˃100 cm (men)	*B*. *animalis* ssp. *lactis* CUL34 (NCIMB 30172)			
4	Lim et. al, 2020^34^	Double‐blind, randomized clinical trial	*n* = 47		*n* = 48	BMI ˃25 kg/m^2^	*Lactobacillus sakei* (CJLS03)	5 × 10^9^ cfu/day	2 assignments of powder per day	12 weeks
			Age (mean) in years 46.4		Age (mean) in years 47.2					
			24.6% men		29.8% men					
5	Michael et. al, 2021^38^	Double‐blind, single‐center, randomized clinical trial	*n* = 35		*n* = 35	BMI 25–29.9 kg/m^2^	*L*. *acidophilus* CUL60 (NCIMB 30157)	5 × 10^10^ cfu/day	1 capsule a day, taken with food, with or without cold beverage	36 weeks
							*L*. *acidophilus* CUL21 (NCIMB30156)			
			Age (mean) in years 52.40		Age (mean) in years 55.26	Waist circumference ˃ 89 cm (women)	*L*. *plantarum* CUL66 (NCIMB 30280)			
							*B*. *bifidum* CUL20 (NCIMB 30153)			
			48.6% women		45.7% women	Waist circumference ˃ 100 cm (men)	*B*. *animalis* subsp. *lactis* CUL34 (NCIMB 30172)			
6	Rahayu et. al, 2021^35^	Double‐blind, randomized clinical trial	*n* = 30		*n* = 30	BMI ≥25 kg/m^2^	*L*. *plantarum* Dad‐13	2 × 10^9^ cfu/day	Little bags, taken after meal	13 weeks
			Age (mean) in years 44.07		Age (mean) in years44.67					
			60% women		60% women					

All the selected studies in this systematic review were randomized, double‐blinded, and placebo‐controlled clinical trials. The studies included a total of 561 individuals, with a mean population age of 47.05 ± 3.48 years. The probiotics used in the included studies belonged to the genera *Lactobacillus* ^34^, ^35^ and *Bifidobacterium* ^36^, ^37^ or a combination of the two.^38^, ^39^ The species employed varied among the studies. For the genus *Bifidobacterium*, the most widely used species was *Bifidobacterium animalis*, specifically the subspecies *lactis* used in four of the trials.^36^, ^37^, ^38^, ^39^ The rest of the species were *Bifidobacterium adolescentis* ^36^ and *Bifidobacterium bifidum*.^38^, ^39^ For the genus *Lactobacillus*, the most widely used species was *Lactobacillus acidophilus* in two of the trials^38^, ^39^ and *Lactobacillus plantar* in three of the trials^35^, ^38^, ^39^; only one trial used *Lactobacillus sakei*.^34^ The mean treatment duration was 18.66 ± 11.65 weeks. The quantities of probiotics used ranged from 1 × 10^10^ colony‐forming units (cfu)/day to 5 × 10^10^ cfu/day. The type of intake varied widely among the various studies: in a fruit milkshake,^37^ with water and without food,^36^ with food,^38^, ^39^ after eating,^35^ and unspecified.^34^ The preparation had 2 different forms: capsules^38^, ^39^ and powders.^34^, ^35^, ^36^, ^37^ The measures employed to assess the treatment efficacy were body weight (kg) and BMI (kg/m^2^) used in all the studies, waist circumference (cm) used in five of the studies,^34^, ^36^, ^37^, ^38^, ^39^ hip circumference (cm) used in one study,^39^ and total body fat (kg) used in two studies.^34^, ^37^

### 3.4 Summary of the results

Table 2 groups the study parameters and results observed in each of the trials.

**TABLE 2:** Parameters studied, evaluation of results, comparison between baseline measures ‐ after treatment and treatment group—placebo.

No	Author and year	Parameter studied	Treatment group				Placebo group				*p*‐value (treat‐ Pbo)
			Baseline measure‐ments (mean ± SD)	Post‐intervention measure‐ments (mean ± SD)	% Change ± SD	*p*‐value	Baseline measure‐ments (mean ± SD)	Post‐intervention measure‐ments (mean ± SD)	% change ± SD	*p*‐value	
1	Stenman et. al, 2016^37^	Body weight (kg)	88.9 ± 10.3	88.49 ± 10.4	NR	NR	88.7 ± 12.5	89.7 ± 12.9	NR	NR	0.15
		BMI (kg/m2)	30.9 ± 1.9	30.8 ± 1.8	NR	NR	31.0 ± 2.2	31.3 ± 2.3	NR	NR	NR
		Waist circumfe‐rence (cm)	103.3 ± 7.5	NR	−2.4	NR	102.1 ± 7.5	NR	NR	NR	0.004
		Hip circumfe‐rence (cm)	NR	NR	NR	NR	NR	NR	NR	NR	0.79
		Total body fat (kg)	35.9 ± 5.2	35.9 ± 5.7	+0.1 ± 7.0	NR	36.4 ± 6.8	37.5 ± 7.1	+3.1 ± 5.6	NR	0.002
2	Krumbeck et. al, 2018^36^	Body weight (kg)	94.8 ± 14.6 (IVS‐1)	NR	−0.1 ± 2.9 (IVS‐1)	NR	96.8 ± 17.7	NR	0.4 ± 5.0	NR	˃0.05
			98.5 ± 32.2 (BB‐12)		0.6 ± 2.4 (BB‐12)						
		BMI (kg/m^2^)	33.9 ± 6.2 (IVS‐1)	NR	−0.1 ± 2.9 (IVS‐1)	NR	34.0 ± 4.5	NR	0.4 ± 5.0	NR	˃0.05
			35.5 ± 10.3 (BB‐12)		0.6 ± 2.4 (BB‐12)						
		Waist circumferen‐ce (cm)	43.5 ± 4.4 (IVS‐1)	NR	0.0 ± 6.9 (IVS‐1)	NR	44.0 ± 11.0	NR	−1.3 ± 3.8	NR	˃0.05
			43.0 ± 9.9 (BB ‐12)		0.2 ± 5.2 (BB‐12)						
		Hip circumferen‐ce (cm)	NR	NR	NR	NR	NR	NR	NR	NR	˃0.05
		Total body fat (kg)	NR	NR	NR	NR	NR	NR	NR	NR	˃0.05
3	Michael et. al, 2020^39^	Body weight (kg)	85.17 ± 13.28	NR	−1.57	<0.0001	83.97 ± 11.68	NR	−0.05	0.7994	<0.0001
		BMI (kg/m2)	29.14 ± 2.73	NR	−1.61	<0.0001	28.97 ± 2.86	NR	−0.07	0.7736	<0.0001
		Waist circumferen‐ce (cm)	100.20 ± 9.04	NR	−0.93	<0.0001	99.52 ± 8.32	NR	−0.01	0.9549	<0.0001
		Hip circumferen‐ce (cm)	NR	NR	NR	NR	NR	NR	NR	NR	NR
		Total body fat (kg)	NR	NR	NR	NR	NR	NR	NR	NR	NR
4	Lim et.al, 2020^34^	Body weight (kg)	73.0 ± 8.6	72.6 ± 8.6	NR	0.352	76.7 ± 10.4	77.2 ± 11.0	NR	0.034	0.058
		BMI (kg/m2)	28.2 ± 2.3	28.0 ± 2.5	NR	0.360	28.5 ± 2.5	28.7 ± 2.7	NR	0.033	0.065
		Waist circumferen‐ce (cm)	91.0 ± 5.6	90.3 ± 5.6	NR	0.017	91.1 ± 7.1	91.3 ± 7.6	NR	0.301	0.013
		Hip circumferen‐ce (cm)	NR	NR	NR	NR	NR	NR	NR	NR	NR
		Total body fat (kg)	27.0 ± 5.1	26.8 ± 5.3	NR	0.454	27.4 ± 5.8	28.0 ± 6.1	NR	0.003	0.018
5	Michael et.al, 2021^38^	Body weight (kg)	83.66 ± 11.26	NR	−4.36	<0.0001	81.16 ± 11.24	NR	−0.60	0.0809	<0.0001
		BMI (kg/m2)	28.10 ± 1.55	NR	−4.38	<0.0001	27.90 ± 1.54	NR	−0.65	0.0628	<0.0001
		Waist circumferen‐ce (cm)	104.20 ± 9.60	NR	−2.91	<0.0001	106.37 ± 11.93	NR	−0.42	0.0521	<0.0001
		Hip circumferen‐ce (cm)	112.40 ± 6.665	NR	−2.58	<0.0001	111.06 ± 7.25	NR	−0.22	0.2789	<0.0001
		Total body fat (kg)	NR	NR	NR	NR	NR	NR	NR	NR	NR
6	Rahayu et.al, 2021^35^	Body weight (kg)	84.54 ± 17.62	83.14 ± 14.71	NR	0.04	79.37 ± 11.76	78.80 ± 11.77	NR	0.12	NR
		BMI (kg/m2)	33.10 ± 6.15	32.57 ± 5.01	NR	0.04	31.80 ± 3.71	31.56 ± 3.67	NR	0.18	NR
		Waist circumferen‐ce (cm)	NR	NR	NR	NR	NR	NR	NR	NR	NR
		Hip circumferen‐ce (cm)	NR	NR	NR	NR	NR	NR	NR	NR	NR
		Total body fat (kg)	NR	NR	NR	NR	NR	NR	NR	NR	NR

The results have been grouped according to the genus of the probiotic employed:

- Genus *Bifidobacterium*, as the only strain

The most widely used strain was *B*. *animalis* subsp. *lactis*. The study directed by Stenman et al.^37^ used the strain *B*. *animalis* subsp. *lactis* 420 as the only probiotic strain at a concentration of 1 × 10^10^ cfu/day. There were significant differences in total body fat mass between the intervention group treated with B420 and the placebo group (*p* = 0.002), which indicates that the probiotic could be effective in controlling body fat, especially in the abdominal region. Despite this difference in fat mass, this significance was not reflected in body weight, although it is certain that the group treated with B420 showed a tendency toward weight reduction compared with the placebo group (*p* = 0.15), the latter gaining approximately 1 kg of weight while the body weight of the treatment group remained stable. There was also a significant difference (*p* = 0.004) of 2.4% in waist circumference between the B420 group (reduction of 2.4 cm) and placebo group.

The trial conducted by Krumbeck et al.^36^ reported the separate use of the strains *B*. *adolescentis* (IVS‐1) and *B*. *animalis* subsp. *lactis* (BB‐12). In this case, there were no significant differences in the anthropometric measurements pre‐ and post‐treatment, although it is important to emphasize the trial's short duration (only 3 weeks) compared to at least 12 weeks for the other studies.

- Genus *Lactobacillus*, as the only strain

Lim et al.^34^ used the strain *L*. *sakei* (CJLS03), reporting in this case a significant decrease in body fat mass (0.2 kg) in the group treated with probiotics and an increase of 0.6 kg in the placebo group (difference of 0.8 kg, *p* = 0.018). The BMI decreased 0.1 kg/m^2^ in the group treated with the probiotic and increased 0.2 kg in the placebo group, without reaching statistical significance (difference of 0.3 kg/m^2^, *p* = 0.065). Body weight decreased 0.3 kg in the probiotic group and increased 0.5 kg in the placebo group (*p* = 0.058). The waist circumference decreased significantly (0.6 cm) in the probiotic group and increased 0.2 cm in the placebo group (*p* = 0.013). In the study conducted by Rahayu et al.,^35^ the employed strain was *Lactobacillus plantarum* Dad‐13. In this case, there were significant differences in the group treated with the probiotic in terms of body weight pre‐ and post‐treatment (*p* = 0.04) as well as in the BMI (*p* = 0.04).

- Combination of the genera *Bifidobacterium* and *Lactobacillus*

The studies conducted by Michael et al.^38^, ^39^ used a combination of the two genera, specifically the following strains: *L*. *acidophilus* CUL60 (NCIMB 30157), *L*. *acidophilus* CUL21 (NCIMB 30156), *L*. *plantarum* CUL66 (NCIMB 30280), *B*. *bifidum* CUL20 (NCIMB 30153), and *B*. *animalis* subsp. *lactis* CUL34 (NCIMB 30172). The trial conducted by Michael et al. in 2020^39^ observed significant differences between the body weight pre‐ and post‐treatment in the group treated with the probiotic (−1.34 kg, *p* < 0.0001), as well as significant differences between the placebo group and treatment group (*p* < 0.0001). There were also significant differences in BMI in the group treated with the probiotic (−1.5%, *p* < 0.0001) in waist circumference (−0.9%, *p* < 0.0001) and in WHI (−1.2%, *p* < 0.0001). There were no significant differences in the placebo group. Weight loss at 3 months was not observed (half of the study). This study also analyzed the quality of life, producing significant improvements in general wellbeing.

The trial conducted by Michael et al. in 2021^38^ also showed statistically significant differences in reducing body weight between the treatment group and placebo group (−3.76%, −3.16 kg, *p* < 0.0001), as well as in body weight pre‐ and post‐treatment in the probiotic group (−4.36%, −3.65 kg, *p* < 0.0001). This weight loss was reflected both in waist circumference (−2.48%, −2.58 cm, *p* < 0.0001) and in hip circumference (−2.36%, −2.66 cm, *p* < 0.0001) in the probiotic group.

## 4 DISCUSSION

The objective of this systematic review was to analyze the efficacy of various probiotics as a therapeutic strategy for treating excess weight and obesity in individuals without associated medical conditions and in the absence of dieting. After the systematic review of the literature of the past 10 years, 6 randomized clinical trials were included that analyzed at least two of the following variables: body weight, BMI, hip circumference, waist circumference and total body fat.

Recently, microbiota that colonize the human gastrointestinal tract have been observed to play an important role in the onset of obesity and other metabolic diseases. Studies have confirmed that this phenomenon is due to the fundamental role played by intestinal microbiota in the host's metabolism, highlighting the regulation of energy homeostasis and its pathogenic role. This statement is based on metagenomic studies that have determined that there are differences in intestinal microbiota between thin individuals and those with obesity.

Changing dietary habits and maintaining an active lifestyle are essential for treating excess weight and obesity; however, the increasingly greater prevalence of this disease indicates that these actions are not easy for the general population. This is particularly due to the difficulty in maintaining body weight in the long term; more than half of the weight lost is regained after 2 years of losing it, and more than three quarters is regained at 5 years.^40^ Given this result, there is an obvious need to investigate new approaches to achieve sustained weight loss over time and prevent its regaining as well as preventing weight gain in individuals with unfavorable environments.

In this review, significant weight reduction was observed in 66.6% of the cases,^34^, ^35^, ^38^, ^39^ and a tendency to weight loss was observed in 16.6%.^37^ Only one study^36^ found no significant differences; however, this was likely due to the short treatment duration compared with the other studies. The results from the included studies indicate a tendency toward preventing weight gain and reducing body weight through the use of probiotics. This effect was most relevant when the genera *Bifidobacterium* and *Lactobacillus* were combined and a variety of strains were employed^38^, ^39^ than when each strain was employed separately. Regardless, the available data indicate that the effect cannot be associated with a specific strain due to the disparity of the strains employed in each study.

As with body weight, BMI declined significantly in 66.6% of the investigated studies, and there was a tendency toward reduction in 16.6% of the studies. The major advantage of this anthropometric measure is the immediacy of its calculation, although it might occasionally be unreliable because it does not consider body fat or muscle mass and could result in errors for certain situations such as athletes with a high percentage of muscle mass. In these trials, however, this measure is considered completely valid because they involve middle‐aged individuals with excess weight and obesity. In this case, the effect is not associated with a single strain, given that the various types employed achieved favorable results; as with body weight, however, superior results appear to be achieved when a combination of genera and strains is employed, rather than when using isolated strains.

Waist circumference is an anthropometric indicator of the concentration of abdominal fat and is widely used in clinical practice to assess visceral fat, enabling the calculation of indices such as the waist‐hip and waist‐height ratios. The waist circumference was analyzed in 5 of the 6 included trials,^34^, ^36^, ^37^, ^38^, ^39^ observing statistically significant reductions in 80.0%.^34^, ^37^, ^38^, ^39^ The strains associated with the effect were *L*. *sakei* (CJLS03), *B*. *animalis subsp*. *lactis* 420, and a combination of various strains of the genera *Bifidobacterium* and *Lactobacillus*.

The anthropometric measurement of hip circumference was analyzed in only one of the trials,^39^ observing statistically significant reductions. Although not considered in most of the studies, hip circumference is highly important as it helps estimate the cardiovascular risk deriving from, in this case, the disorder of obesity or excess weight. The WHI could be associated with a combination of strains from the genera *Bifidobacterium* and *Lactobacillus*.

Total body fat mass was analyzed in only two^34^, ^37^ of the six trials, finding statistically significant reductions in both. The effect was observed with the strains *B*. *animalis subsp*. *lactis* 420 and *L*. *sakei* (CJLS03).

It should be noted that this systematic review has been carried out based on clinical trials and randomized controlled trials in humans with no associated medical conditions apart from obesity or overweight (including hypertension, diabetes, metabolic syndrome, Prader‐Willi syndrome, cancer, etc.) and in the absence of diet, which significantly reduces the number of clinical trials included in the study. This fact has also been observed in the few similar reviews prior to the one presented,^41^, ^42^, ^43^, ^44^ which do not include the last 5 years taken into account in the present review. Park and Bae^42^ observed, in a systematic review about probiotics for weight loss, that collectively, the probiotics had limited efficacy in terms of decreasing body weight and BMI and were not effective for weight loss. On the other hand, Borgeraas et al.,^41^ in a systematic review and meta‐analysis conducted to examine the effects of probiotic supplementation on body weight, BMI, fat mass and fat percentage in subjects with overweight or obesity, observed a significantly larger reduction in body weight, BMI and fat percentage, compared with placebo, results in accordance to observed by Wang et al.^44^ which concluded that the probiotic supplementation could potentially reduce the weight gain and improve some of the associated metabolic parameters; Similarly, Tomé‐Castro et al.^43^ observed that some probiotic strains are shown to be effective in reducing BMI and hip circumference. With regard to our results and in accordance with the referred revisions, there are few human randomized clinical trials to reach a conclusion regarding the efficacy of using probiotics as effective preventive measures in obesity and excess weight. The various trials performed with humans have had mixed results, which can be attributed to their methodological diversity, low homogeneity of the study population, varying sample sizes, disparity of strains studied, and short intervention time employed. These observations agree with those reported by other authors who have highlighted the disparity of the results.^41^, ^42^, ^45^ Thus, more rigorously designed randomized clinical trials are necessary to examine the effect of probiotics on weight loss, reduction in BMI, WHI, or waist circumference in greater detail.

The present systematic review confirmed that the strains *B*. *animalis subsp*. *lactis* 420 and *L*. *sakei (CJLS03)* in isolation have shown consistent results toward improving the parameters related to controlling weight, including body weight, total fat mass, BMI, and waist circumference. The results also indicate that a combination of strains of the genera *Bifidobacterium* and *Lactobacillus* favor this improvement.

Several recently published studies^40^, ^41^, ^43^, ^46^, ^47^, ^48^ have reported favorable results in this area. Studies have reported that the use of certain species such as *Streptococcus thermophilus*, *Lactobacillus bulgaricus*, and *L*. *acidophilus* is a valid strategy for treating excess weight and obesity.^43^ According to our study's results, the studies of the systematic review with meta‐analysis have shown a reduction in body weight and percentage body fat^41^ as well as in BMI and waist circumference.^46^ All of the studies agree that there is a positive tendency in improving the anthropometric parameters through probiotic supplementation; however, they highlight the need for more studies to overcome the current limitations. One of the conclusions is that future clinical trials should focus on long‐term therapies, given that a clear relationship has been observed between treatment duration and beneficial effects. It will also be important to standardize the strains and their concentrations to be able to establish solid recommendations for clinical practice and procedure homogeneity.

The possible mechanism that has been proposed to explain the results is that probiotics would help restore homeostasis of the intestinal microbiome and therefore the human metabolic homeostasis at the gut level, which are altered in overweight and obesity. The probiotic supplementation might increase directly or indirectly through cross‐feeding substrates and health‐promoting metabolites. An example has been recently reported by our research group,^49^ in an ex vivo study that investigated the effects on the composition and metabolic activity of the intestinal microbiota of three probiotic‐based food supplements, containing, respectively, (1) *Bifidobacterium longum* ES1, (2) *Lactobacillus acidophilus* NCFM®, and (3) a combination of *L*. *acidophilus* NCFM®, *Lactobacillus paracasei* Lpc‐37™, *Bifidobacterium lactis* Bi‐07™, and *Bifidobacterium lactis* Bl‐04™. It was found that the combined formulation probiotics, in agreement with reported in the present systematic review, significantly impacted the intestinal microbiome, showing, as the health benefit endpoint, a significant stimulatory effect on bifidobacteria and lactobacilli growth and changes in metabolic activity characterized by the significant stimulation of short chain fatty acids and branched short‐chain fatty acids (BCFAs) and ammonium. These microbiota‐derived metabolites produced by the fermentation of dietary fiber and amino acids, respectively, might influence key aspects of inflammation and gut barrier permeability. In addition, probiotics might demonstrated extra‐intestinal effects by helping the communication axis among gut microbiota and the organs via neural, endocrine, immune, humoral, and metabolic pathways.^5^ Probiotics might play a significant role in digestion, metabolic processes (e.g., the regulation of cholesterol absorption, blood pressure, and glucose metabolism), related to the clinic of overweight and obesity.^6^, ^7^, ^8^

Finally, the presented systematic review has various limitations, including the heterogeneity of the included studies. The language bias also needs to be considered, given that only those articles published in English were included. A considerable majority of the studies found in the literature search were based on the change produced in the intestinal microbiota by the disease and after the intervention. Many of the studies neglected the anthropometric measurements, which prevented the use of numerical data from most of the selected studies to perform a meta‐analysis. The heterogeneity of the trials also hindered their comparison.

## 5 CONCLUSIONS

This review provides evidence of a trend in preventing body weight gain and reducing weight through the use of probiotics in individuals with excess weight or obesity. The efficacy of certain probiotic strains has been verified, especially the combination of various strains of the genera *Bifidobacterium* and *Lactobacillus* in changing anthropometric parameters such as body weight, waist circumference, and total body fat in individuals with excess weight and obesity. Greater homogeneity in the procedures is needed in future clinical trials to be able to establish clear recommendations for clinical practice.

## AUTHOR CONTRIBUTIONS

Authors' contributions are indicated in the body of the article.

## CONFLICT OF INTEREST STATEMENT

The authors declare that the research was conducted in the absence of any commercial or financial relationships that could be construed as a potential conflict of interest.

## CONSENT FOR PUBLICATION

The authors declare their consent for publication.

## Data Availability

Send requests to the corresponding author.
